# Saving lives through certifying deaths: assessing the impact of two interventions to improve cause of death data in Perú

**DOI:** 10.1186/s12889-018-6264-1

**Published:** 2018-12-03

**Authors:** Janet Miki, Rasika Rampatige, Nicola Richards, Tim Adair, Juan Cortez-Escalante, Javier Vargas-Herrera

**Affiliations:** 1 Civil Registration and Vital Statistics, Vital Strategies, Bloomberg Data for Health Initiative, Santiago de Surco, Lima, Peru; 2 Melbourne School of Population and Global Health, The University of Melbourne, Carlton, Victoria Australia

**Keywords:** Cause of death, Certification, Intervention, Mortality, Online, Perú, Quality, Training

## Abstract

**Background:**

Mortality statistics derived from cause of death data are an important source of information for population health monitoring, priority setting and planning. In Perú, almost all death certificates are issued by doctors because it is a legal requirement. However, the quality of cause of death data is poor. In August 2016, the Ministry of Health of Perú decided to make two specific interventions to improve cause of death data: to introduce an online death certification system and to train doctors in standard death certification practices.

**Methods:**

The study comprised a random sample of 300 pre-intervention death certificates, 900 death certificates that were part of the online intervention, and 900 death certificates that were part of both the online and training interventions. All the deaths had occurred between January and September 2017. We used the Assessing the quality of death certification tool from the University of Melbourne for the assessment. We examined the frequency of common errors in death certificates, the frequency of any error and the average error score for each category of: age group, sex, doctor’s seniority, doctor’s speciality, level of health facility and broad cause of death.

**Results:**

The average error score declined by 38% due to the online intervention and by a further 26% due to the training intervention. Improved certification practices remained after controlling for potentially confounding factors. Main improvements were reductions in the absence of a time interval (66% of certificates), incorrect sequence of causes (22%), and ill-defined conditions (13%).

**Conclusions:**

This study demonstrates how the two interventions introduced by the Ministry of Health in Perú improved the correctness of death certificates. The study also provides evidence on necessary changes to the training program to address the poor certification practices that have remained after implementation of the online system.

**Electronic supplementary material:**

The online version of this article (10.1186/s12889-018-6264-1) contains supplementary material, which is available to authorized users.

## Background

Routine cause of death data from civil registration and vital statistics (CRVS) systems are an important source of information and evidence for monitoring population health, identifying health priorities, and planning public health interventions [1–4]. However, many countries have registration systems that cover only part of the population, with no cause of death data for those dying outside of health facilities, and no routine compilation of data for analysis, dissemination, and policy purposes. Furthermore, many hospitals’ cause of death data, derived from medical certification, are of such poor quality to be not useful for policy making [5–10]. There are many reasons for the poor quality of death certification, including: i) absent, insufficient, or inadequate training on death certification and the concept of underlying cause of death, ii) limited understanding of the importance of death certification within the health sector, iii) an absence of doctors in some rural areas to certify deaths, iv) challenges in obtaining cause of death for external causes, v) doctors’ experience in the certification of deaths, and vi) limited availability of diagnostic equipment [11–13].

In 2016, approximately 145,500 deaths were registered in Perú, with registration completeness estimated at 75% [14]. Coverage is a major challenge for the country, with 89% of deaths registered in offices of the Civil Registry located in urban areas, and only 11% registered in municipalities, despite approximately one-quarter of the population living in rural areas [15]. While 95% of medical certificates of cause of death (‘death certificates’) are certified by doctors [16], international studies have shown that cause of death data from certificates are of limited use for country policy due to their poor quality [6, 17, 18]. Such findings are also supported by Perú’s low Vital Statistics Performance Index (VSPI) score (50%). The VSPI scores the quality of data on mortality and cause of death by assessing several components within a CRVS system, including the extent to which age and/or sex are missing in the data, and the number of biologically implausible underlying causes of death recorded on death certificates [19].

One reason for the poor quality of cause of death data in Perú is the poor quality of medical certification. Medical certification of cause of death is not a primary focus of the undergraduate medical curriculum in Peruvian universities, with the current curricula mostly focusing on prevention, and the recuperation and rehabilitation of patients. Many doctors do not understand the link between the causes they record on death certificates and national mortality statistics. Further, many doctors are not aware of standard death certification guidelines and practices. These factors all affect the quality of cause of death data reported in Perú [20–22].

To address these issues the Ministry of Health (MOH) of Perú, with the help of the Bloomberg Philanthropies Data for Health Initiative, introduced two specific interventions to improve the completeness of death registration, and the quality of cause of death data. The first intervention, in August 2016, was to introduce an online death notification and certification system (SINADEF^1^) to all health facilities and morgues. SINADEF is modelled on the system of online birth certification introduced by the MOH in 2012, which saw birth registration increase from 67% in 2011 to 84% in 2017 [23–25]. The introduction of online death certification was also supported by evidence from other countries that had shown improvements in the completeness of death notification and registration following their implementation [26, 27].

SINADEF was introduced to improve the processes of death notification and certification through providing a secure data entry and storage system. The system also improved the quality of cause of death data entered through: i) eliminating the effect of illegible handwriting; ii) reducing the use of ill-defined conditions by having a warning pop up dialog (for ‘cardiac arrest’ only); iii) not allowing for blank spaces to be left between the lines of events; and iv) decreasing the use of abbreviations, due to the high number of characters (300) allowed on each line. Given its online nature, SINADEF also improved the timeliness of data, as online certificates are available immediately to the MOH.

To ensure maximum benefit from SINADEF, the MOH also introduced a training program to doctors on medical certification, including how to complete the International Form of Medical Certificate of Cause of Death [28] correctly. A selected team of trained doctors and other professionals trained a group of doctors and statisticians from the main hospitals and morgues in how to use SINADEF, as well as a group of regional staff to replicate the training in other health facilities.

The training lasted two hours: one hour on the online system and one hour for certification of cause of death. The training was limited to two hours because all health facilities considered it was the maximum time that doctors could be released from clinical duties. The following topics were covered during the training: the importance of correct medical death certification of cause of death; understanding the International Form of Medical Certificate of Cause of Death; the concept of underlying cause of death (UCOD); case scenarios to demonstrate correct certification practices; and SINADEF.

Previous studies have shown the effectiveness of knowledge gained in medical certification training based on pre-and post-training tests using clinical scenarios. However, most of these studies have been conducted as one-off trainings with little or no follow up, and literature on the effectiveness of medical certification training producing sustainable changes to death certification practices is rare [29, 30]. The literature has shown the importance of periodically monitoring death certification accuracy to identify quality issues to provide feedback to certifiers and their administrators to improve them [31, 32].

To assess the effectiveness of the two interventions introduced by the MOH to increase the quality of cause of death data, in this study we compare the quality of medical certification correctness between three groups:

1. Paper death certificates completed prior to the two interventions (‘pre-intervention’)
2. Death certificates entered in SINADEF by doctors (‘online intervention’)
3. Death certificates entered in SINADEF by doctors who had also received medical certification training (‘online and training intervention’).

This study was carried out to evaluate the quality of cause of death data in Perú, as generated from medical certificates of cause of death completed by doctors. The results will provide feedback to doctors and their administrators about current routine medical certification correctness to identify areas that need further training, and provide recommendations to the MOH’s medical certification training program.

## Methods

A random sample of 300 pre-intervention, 900 online intervention, and 900 online and training intervention death certificates, completed between January 2017 and September 2017 were selected for the study. Doctors were unaware they were being evaluated before and after the intervention. All online death certificates were obtained from SINADEF.

For the assessment, the tool *Assessing the quality of death certificates* from the University of Melbourne was used [33]. This tool is designed to assess the quality of death certification practices by checking for the presence of common errors in death certificates. The tool can also be used to assess the quality of death certification as part of routine assessment, or to assess the training needs of doctors in designing cause of death certification training and to evaluate the effect of death certification training. Errors were classified as major or minor errors, as shown in Table 1; and a summary error score was developed with two points applied to major errors and one point for minor errors.

**Table 1:** Classification matrix of major and minor errors assessed in death certificates

Error type	Description and implications
Major errors	
Multiple causes per line	The WHO ICD guidelines state that only one cause should be recorded per line in a death certificate. When more than one cause is reported on a single line, it makes it difficult for coders to establish the sequence of events leading to death, thus selecting the correct underlying cause of death would be more difficult
Absence of disease time interval	The time interval should be entered for all conditions reported on the death certificate, especially for the conditions reported in Part 1. Time intervals are very important for correctly coding certain diseases and provide a check on the accuracy of the reported sequence of conditions.
Incorrect sequence of events leading to death	Mortality statistics are based on the underlying cause of death, which is the condition or injury that initiated the sequence of events that led directly to death. When a clinically improbable sequence of events is recorded, it is impossible to select the correct underlying cause of death.
Ill-defined or poorly specified condition entered as the UCOD	Ill-defined or poorly specified conditions are of no value for public health officials, and do not provide any information for decision-makers to help them design preventive health programs. These include, for example, organ failure (hepatic or cardiac failure, etc.); symptoms or signs (hematemesis, dyspnoea, fever, etc.); mode of dying (cardiac arrest, respiratory arrest); pathophysiological findings (shock); other (trivial diseases such as colds, rhinitis, etc.).
Minor errors	
Presence of blank spaces within the sequence of events	In completing a death certificate the certifier should use consecutive lines in Part 1 of the death certificate starting at Line 1a. The UCOD should be recorded in the lowest used line of Part 1. There should not be any blank lines within the sequence/chain of events leading to death.
Abbreviations used in certifying the death	Doctors are encouraged not to use abbreviations when certifying deaths as abbreviations can mean different things to different people. There is a chance that coders may misinterpret the abbreviation and code the death to a non-relevant code.
Additional errors on the certificate	There may be other additional errors on death certificates including: incomplete information of the external cause of death (no site of the injury, intent or nature of it, etc.); insufficient information on neoplasms (no site, whether benign or malignant, etc.); failing to identify pregnancy and maternal deaths.

The errors were categorized as major or minor based on the impact that the error can have on the final selection of underlying cause of death (UCOD) by the mortality coder. If the risk of mis-identification by coders is high, the error was classified as a major error. For example, recording multiple causes per line makes it difficult for coders to apply selection and modification rules for selecting the UCOD. On the other hand, presence of blank lines has a minor impact on the process of UCOD selection, and so was classified as a minor error.

### Inclusion and exclusion criteria

The study was conducted in almost all departments (24 out of 25, one department was excluded because of an unreliable internet connection and doctors’ resistance to use the online system) that certify deaths using SINADEF in Perú. The study death certificates entered in SINADEF directly by certifying doctors, and certificates entered by trained statisticians. To minimize other factors that may influence the quality of certification, the selection excluded death certificates from doctors who were not the attending doctor for the deceased, and death certificates that were registered outside a health facility, as the doctors there may not have sufficient technology or information to correctly complete the death certificate.

### Study procedures

Data from selected death certificates (online certificates completed by doctors and paper death certificates manually entered in SINADEF by statisticians) were downloaded. The data included general information on the deceased including age, sex, place and name of health facility where the person died; and certifier data such as doctor’s name and Medical Council registration number. The name and Medical Council registration number of doctors were matched with those from attendance lists at SINADEF training, to identify certificates for the ‘online and training intervention’ group. The name of health facility was matched with information from the National Health Superintendence (SUSALUD) to select the level of the facility (I-III). To complete the data about the doctor’s seniority and speciality, information from the Medical Council was used.

The evaluation of death certificates was carried out by one doctor with experience in death certification who received a training in using the death certification assessment tool that was conducted as part of the implementation of SINADEF.

### Data analysis

Bivariate and multivariate analyses were conducted to assess the effectiveness of the three intervention groups. Bivariate relationships were analysed using chi-square and t-tests. The multivariate analysis conducted was an ordinal logit regression of the error score, to identify if the interventions had a statistically significant impact on the quality of death certification (measured by the error score), controlling for potentially confounding factors. The covariates in the model were study group status (pre-intervention, online intervention, online and training intervention), age group of the deceased, sex, doctor’s experience, doctor’s specialisation, level of health facility, and cause of death (as classified into Global Burden of Disease [GBD] group). Doctor’s experience and specialisation and the level of health facility may differ between the three stages of the study, and their inclusion in the regression removes any bias they may introduce into the results for the interventions. An ordinal logit is suitable when the dependent variable is ordered, such as the error score, which ranged from 0 to 9 [34]. The ordinal logit regression produces odds ratios; these show the odds of a variable category having a higher error score compared with the reference category for that variable, controlling for all other variables in the regression. The data were analysed using Stata 15 [35].

## Results

### Study population

From January to September 2017, 22,727 deaths were certified in SINADEF and 3298 deaths were originally certified using a paper certificate and then transcribed into the system. Of these records, 15,656 (69%) deaths occurred in a health facility and the other 7071 deaths occurred away from health facilities, including at home, at work, etc. A total of 2517 doctors certified the 15,656 deaths that occurred in a health facility, with 1257 doctors certifying the 2100 deaths from our random sample (approximately 50% of all doctors using SINADEF). The most common level of doctor’s experience was 11–15 years across all study groups. More information on attributes of the death certificates assessed are provided in Additional file 1.

### Correctness of death certificates

Table 2 shows that the correctness of death certification was much higher following the interventions, increasing from 0 to 30% due to the introduction of the online system, and then further to 43% with the online system and training. The correctness at pre-intervention was 0% as the clear majority (96%) of certificates did not have the time interval recorded. When this error type is removed, the correctness of pre-intervention certificates increases to 15%.

**Table 2:** Correctness of death certificates assessed

	Correctness of death certificates according to study group (%)			Percentage point improvement (%)	
	Pre-intervention (*n* = 300)	Online intervention (*n* = 900)	Online and training intervention (*n* = 900)	Online intervention	Online and training intervention
Correctly certified certificates	0.0	30.1	43.3	30.1**	43.3**
Certificates with one error	16.3	18.2	18.6		
Certificates with two errors	40.0	28.0	22.9		
Certificates with three errors	27.3	17.9	12.8		
Certificates with four or more errors	16.3	5.8	2.4		
Total (%)	100.0	100.0	100.0		

**p < 0.01

**Fig. 1:**
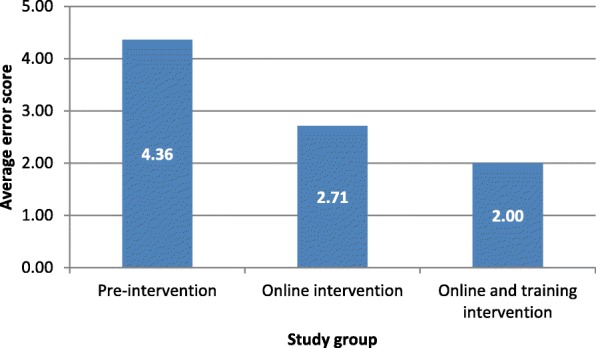
Average error scores of death certificates by study group

The composite error score declined by 38% due to the online intervention, and a further 26% due to the online and training intervention (Fig. 1).

The most important improvement in the type of error was in the time interval of the disease, with 66% of the online and training intervention group certificates having this correctly completed. There was also an important improvement (22%) in the correctness of the sequence of events leading to death, and in no longer reporting ill-defined conditions as the underlying cause of death (13%) (Table 3).

**Table 3:** Major and minor errors in death certificates assessed

Error type	Correctness of death certificates according to study group (%)			Percentage point improvement (%)	
	Pre-intervention (*n* = 300)	Online intervention (*n* = 900)	Online and training intervention (*n* = 900)	Online intervention	Online and training intervention
Major errors					
Multiples causes per line	2.0	1.3	0.6	0.7	1.4
Absence of disease time interval	96.0	47.1	30.0	48.9**	66.0**
Incorrect sequence of events leading to death	40.3	25.9	17.9	14.4**	22.4**
Ill-defined condition entered as an underlying cause of death	52.0	45.4	38.9	6.6*	13.1**
Minor errors					
Presence of blank lines within the sequence of events	11.3	0.2	0.3	11.1**	11.0**
Abbreviations used in certifying the death	11.7	4.6	4.1	7.1**	7.6**
Additional errors on the certificate	32.3	26.6	21.0	5.7	11.3**
- External causes, missing details	4.7	4.6	2.1		
- Neoplasms, missing details	15.0	8.1	6.3		
- No units in age of deceased	0.0	0.2	0.6		
- Other	12.7	13.7	12.0		

* p < 0.05 ** p < 0.01

The occurrence of additional errors in death certificates decreased from 32% in the pre-intervention group, to 21% in the online and training intervention group (Table 4). Within this error type, ‘other’ included non-specification of hypertension as essential or secondary, non-specification of diabetes as type I or II, and recording the UCOD in Part 2 of the death certificate. There was no change in the number of certificates with these ‘other’ errors when comparing pre-intervention with online and online and training interventions.

**Table 4:** Certificates with any error and their average error score, according to attributes of the deceased and certifier

Attributes of the deceased	Any error (%)	Average error score	Number of death certificates
Age group (years)			
0–4	50.2	1.59	194
5–44	65.7	2.65	248
45–64	64.9	2.47	373
65–74	71.5	2.76	368
75–84	72.9	2.85	516
≥ 85	74.6	2.92	394
Sex			
Male	69.9	2.68	1087
Female	66.8	2.60	1007
Cause of death (GBD categories)			
Communicable diseases	68.9	2.69	730
Non-communicable diseases	64.7	2.35	1175
External causes	87.7	3.93	71
Ill-defined (unusable and insufficiently specified) causes of death	100.0	5.00	124
Attributes of the certifier	Any error (%)	Average error score	Number of death certificates
Doctor’s seniority (years)			
0–5	80.9	3.17	141
6–10	71.4	2.78	405
11–15	62.6	2.38	412
16–20	73.2	2.93	370
21–25	65.3	2.41	245
26–30	62.8	2.41	239
> 30	68.4	2.57	288
Doctor’s speciality			
General medicine	69.0	2.71	468
Internal medicine	67.4	2.58	746
Pediatrics & neonatology	52.1	1.62	165
Intensive care medicine	73.6	3.08	178
Pneumology	63.2	1.93	57
Oncology	55.6	2.33	36
Emergency	85.9	3.52	170
General surgery	74.6	2.75	63
Others	67.7	2.62	217
Level of health facility			
I (health centre)	91.4	3.86	35
II (hospital)	68.8	2.66	982
III (specialised hospital)	67.5	2.58	1083
Time from training to death certification^+^			
< 3 months	54.7	1.87	234
3–6 months	60.4	2.17	455
> 6 months	50.7	1.76	211

^+^Does not include certificates from the pre-intervention study group

Table 4 shows that the percentage of death certificates with any error and the average error score increased with age of the deceased. While there was no linear decrease in the number of errors and average error score as doctor’s seniority increased, overall, junior doctors (with 0–5 years’ experience) had the highest number of certificates with any error reported (81%) and the highest average error score (3.2). Level II and III hospitals are generally located in urban areas, while level I health centres are located in rural areas; certificates with the highest error score included those from Level I health facilities, those with unusable or external causes, and those certified by doctors specialising in emergency or intensive care medicine. For the period between receiving training and completing the death certificate, the highest error scores were from doctors that certified between 3 and 6 months following the training.

Table 5 shows the results from an ordinal regression of the error score of the pre-intervention, online intervention, and online and training intervention groups. Introduction of the online system resulted in a death certificate having smaller odds of having a higher error score compared with pre-intervention (0.283). For certificates in the online and training intervention, there was only 0.151 the odds of having a higher error score compared with pre-intervention. This means that certificates in the online and training intervention had 6.6 times greater odds (1/0.151) of having a lower error score compared with pre-intervention certificates.

**Table 5:** Results of ordinal regression of the error score

Variables	Odds Ratio	Std. Error	P > │z│
Study group			
Pre-intervention	Ref.	–	–
Online intervention	0.283**	0.034	0.000
Online and training intervention	0.151**	0.019	0.000
*Online and training* versus *online intervention*^*+*^	0.535**	*0.048*	*0.000*
Attributes of the deceased			
Age group (years)	1.009**	0.002	0.000
Sex			
Male	Ref.	–	–
Female	0.969	0.077	0.673
Cause of death (GBD categories)			
Ill-defined (unusable and insufficiently specified) causes of death	Ref.	–	–
Communicable diseases	0.181**	0.036	0.000
Non-communicable diseases	0.120**	0.024	0.000
External causes	0.486*	0.138	0.011
Attributes of the certifier	Odds Ratio	Std. Error	P > │z│
Doctor’s seniority (years)			
0–5	Ref.	–	–
6–10	0.825	0.151	0.295
11–15	0.591**	0.109	0.004
16–20	0.967	0.187	0.864
21–25	0.713	0.143	0.091
26–30	0.783	0.160	0.231
> 30	0.782	0.154	0.210
Doctor’s speciality			
General medicine	Ref.	–	–
Internal medicine	1.134	0.133	0.285
Pediatrics & neonatology	0.877	0.190	0.544
Intensive care medicine	1.495*	0.250	0.016
Pneumology	0.725	0.189	0.217
Oncology	0.753	0.263	0.417
Emergency	2.023**	0.340	0.000
General surgery	1.057	0.260	0.822
Others	1.154	0.185	0.370
Level of health facility			
I (health centre)	Ref.	–	–
II (hospital)	0.670	0.216	0.214
III (specialised hospital)	0.757	0.246	0.391

* p < 0.05 ** p < 0.01

^+^This result used “online intervention” as the reference category; all regression results for other variables were the same as when reference category is “pre-intervention”

*Ref.* Reference category

Additional analysis shows that the introduction of the training intervention resulted in significantly smaller odds (0.535) of having a higher error score compared with the online intervention only. Regarding attributes of the deceased, the odds of having a higher error score increased with age, and this was statistically significant.

## Discussion

This study has clearly demonstrated the effect of the two interventions introduced by the Ministry of Health in Perú to improve the correctness of cause of death data. All pre-intervention death certificates had at least one error and with introduction of the online system SINADEF, one-third of them were improved. After introducing the training program to complement SINADEF, almost half of the death certificates were improved. The random sample of death certificates used in this study included about half of all doctors that are using the online system; making the results of the data assessment more generalisable.

### Effect of the online system

Overall, introduction of the online system for death certification had the greatest impact on improving the quality of death certification; with the training program producing modest further improvements. There have been previous studies in France [27, 28], where researchers compared the quality of paper and electronic death certificates and found that doctors complete the electronic death certificates better than the paper ones. This study reinforces these findings. Having an electronic certificate with built-in requirements reduces the number of errors as, for example, doctors can’t leave blank spaces between the sequences of events as the system does not allow it. The use of abbreviations also decreased when using SINADEF, likely due to the high numbers of characters allowed (300) in each cause of death line, unlike the short space in paper death certificates. Also, entering an ill-defined condition as the UCOD decreased with SINADEF, likely as the system opens a warning window when the doctor records an ill-defined condition (such as cardiac arrest) indicating that it isn’t an UCOD.

While establishing an online system for death certification or any other part of the certification and registration process is a major undertaking, many countries are already implementing various electronic technologies as part of their CRVS systems. For developing countries especially, the innovative use of technology is allowing them to ‘leap-frog’ the traditional development pathway and achieve substantial gains [36]. This study further demonstrates the usefulness and feasibility of introducing an electronic system as part of routine CRVS processes.

### Effect of the online system and training

Training in medical certification of cause of death has showed improvements in the correctness of certification at a routine level. Several studies have shown different levels of improvements with training [28, 29, 37–39]. While the addition of training did lower the average error score when compared with the online intervention alone, the impact was not substantial. This may be because subsequent improvements in certification are harder to achieve, after initial gains when implementing an online system. The limited improvement in the correctness of death certificates may also be due to:

- Inadequacy of the training program (time or content) [28].
- The change in knowledge (gained through training) does not produce significant change in certification behaviour at the practice level.
- Inadequate assessment of death certification correctness and feedback to the certifiers.
- The disjointed nature of training, highlighting the need to ensure continuous training to medical students, junior doctors, and experienced specialists [37, 40, 41].

After implementation of the online system, one-third of the remaining ill-defined conditions entered as an underlying cause of death were in the category of an intermediate cause [42]. This could mean that doctors don’t have further information to identify the UCOD; perhaps due to limited diagnostic capacities of the hospital, or that they were not aware of the importance of identifying and reporting the correct UCOD for public health. Additional research comparing death certificates with the medical reports would be necessary to identify the reason for the high proportion of intermediate cause of death. Additional errors in the death certificates included insufficiently specified causes, and listing an incorrect sequence of events leading to death. Future training programs should focus on these errors.

There are some limitations to our study. First, the study compares trained doctors versus non-trained ones as per the recent training by the MOH. We do not have information regarding any previous training exposures among the pre-intervention and the online intervention groups. However, we are assuming that most of the doctors did not have access to good quality training. We also did not have information on the frequency that trained doctors certify deaths, though the literature shows mixed results when comparing level of experience with correctly certified deaths [40, 43, 44]. A quality assessment with the same group of doctors pre- and post-training would provide better information. Second, it was observed that, despite being mandatory, older doctors were more likely to resist using the electronic system, which may have affected the results. However, doctor’s seniority was controlled for in the regression analysis and it was not found to be significant (apart from 11 to 15 years experience) so any impact of this effect is likely to be minimal. Third, many level I facilities are in remote rural areas lacking reliable access to the Internet, and so they were excluded from the study. However, the number of deaths in such facilities is minimal compared with those from levels II and III. A final limitation is the potential for introduction of random and systematic errors by only having one doctor review the certificates. However, the use of a single reviewer eliminated any inter-interviewer variability; the sample of certificates were reassessed by an experienced reviewer at the end of the study; and research has demonstrated that single, rather than duplicate, review processes do not lead to significant diagnostic inaccuracy, as previously assumed [45].

## Conclusions

The study has demonstrated the significant impact that an online system can have on the quality of death certification, which in Perú will result in improved mortality statistics as the system is rolled out through the country. Online certification should be developed in other countries to improve not only completeness of registration, but also data quality. The lessons learned in Perú around the design and implementation of SINADEF hold important insights for other countries looking to implement similar systems: making key variables mandatory (for example, the time interval between disease onset and death, and not allowing blank lines when entering the sequence of events leading to death) would automatically improve the quality of death certificates. Similarly, having warning windows appear on-screen when a doctor attempts to enter an ill-defined condition as an underlying cause of death would likely lead to a decrease in such behaviour, and an increase in correctly coded mortality statistics.

The addition of medical certification training did not produce as large a change in death certification correctness as the introduction of SINADEF, possibly because the improvements due to SINADEF were relatively easier to achieve (i.e. inclusion of time interval on certificate). Again, countries wishing to improve the quality of their cause of death statistics can learn from the experiences of Perú and may look to: adapt their training programs to focus on certification errors that remain after the introduction of certification interventions (such as online systems or periodic reviews); introduce a master training program at the health facility level, where local trainers can provide training sessions on a regular basis; introduce certification training in medical curriculum, to ensure doctors receive adequate time on the subject as part of their education; and implement periodic death certificate assessments for further improvement of certification correctness. For the sustainability of periodic assessments, we recommend including training in assessing the quality of death certificates to a small team of health professionals in each department/health facility, so that they can do the follow up, bring feedback periodically, and retrain doctors. This team could analyse the main errors in the different health facilities of the department and change the content of the training for each place according to the results of the analysis.

In looking to the future for Peru, the next major step is to continue to roll-out implementation of Iris, the automated coding software. A team has been established within the Ministry of Health to develop a Peruvian version of the dictionary, and has re-coded 2016 data using Iris. Initial results have been very positive, with Iris able to code over 70% of medical certificates. The remaining certificates were analysed by a team of statistical personnel from the Ministry of Health and Directorates of Integrated Health Networks in Lima and Callao. In the coming months, 2017 data will also be analysed, as the country continues to move to electronic solutions for improving the quality of its mortality data.

## Additional file

Additional file 1: Attributes of death certificates assessed. (DOCX 13 kb)

## Abbreviations

CRVS: civil registration and vital statistics
MOH: Ministry of Health, Perú
SINADEF: Sistema Informático Nacional de Defunciones (National Death Information System)
UCOD: underlying cause of death
VSPI: Vital Statistics Performance Index

## Footnotes

1 SINADEF is the abbreviation in Spanish of Sistema Informático Nacional de Defunciones (National Death Information System).
