# Supplementary material for: Saving lives through certifying deaths: assessing the impact of two interventions to improve cause of death data in Perú
Source: BMC Public Health. 2018 Dec 3;18:1329. doi: 10.1186/s12889-018-6264-1 (PMC6276144; doi:10.1186/s12889-018-6264-1)
Supplement: Supplementary file 1 — Attributes of death certificates assessed. (DOCX 13 kb) [file 12889_2018_6264_MOESM1_ESM.docx]

|  | **Attributes of death certificates assessed (%)** | | | | |
| --- | --- | --- | --- | --- | --- |
| **Attributes of the deceased** | | | | | |
| **Age group (years)** | **Pre-intervention**  **(n=300)** | **Online intervention**  **(n=900)** | **Online and training intervention**  **(n=900)** | **All study death certificates**  **(n=2100)** | **All online death certificates**  **(n=22627)** |
| 0-4 | 6.0 | 9.0 | 10.6 | 9.6 | 7.3 |
| 5-44 | 10.7 | 12.6 | 11.4 | 11.9 | 16.7 |
| 45-64 | 15.0 | 19.3 | 17.1 | 17.7 | 20.5 |
| 65-74 | 18.0 | 17.7 | 17.2 | 17.4 | 16.0 |
| 75-84 | 28.7 | 23.9 | 23.9 | 24.4 | 21.0 |
| ≥85 | 21.7 | 17.3 | 19.2 | 18.6 | 17.9 |
| Not specified | 0.0 | 0.2 | 0.6 | 0.5 | 0.6 |
| **Sex** | | | | | |
| Male | 50.0 | 53.1 | 51.0 | 51.8 | 55.6 |
| Female | 50.0 | 46.7 | 48.6 | 47.8 | 44.0 |
| Not specified | 0.0 | 0.2 | 0.4 | 0.4 | 0.4 |
| **Cause of death (GBD categories)** | | | | | |
| Communicable diseases | 32.0 | 34.9 | 37.0 | 35.4 | 35.4 |
| Non-communicable diseases | 58.3 | 55.9 | 57.2 | 56.8 | 57.8 |
| External causes | 3.0 | 4.7 | 2.5 | 3.5 | 3.1 |
| Ill-defined (unusable and insufficiently specified) causes of death | 6.7 | 4.5 | 3.3 | 4.3 | 3.7 |
| **Attributes of the certifier** | **Pre-intervention**  **(n=300)** | **Online intervention**  **(n=900)** | **Online and training intervention**  **(n=900)** | **All study death certificates**  **(n=2100)** | **All online death certificates**  **(n=22627)** |
| **Doctor's seniority (years)** | | | | | |
| 0-5 | 14.3 | 4.7 | 6.2 | 6.7 | 9.7 |
| 6-10 | 19.7 | 21.7 | 16.8 | 19.3 | 20.2 |
| 11-15 | 22.0 | 20.7 | 17.8 | 19.6 | 20.8 |
| 16-20 | 12.0 | 20.2 | 16.9 | 17.6 | 16.5 |
| 21-25 | 12.7 | 9.4 | 13.6 | 11.7 | 11.6 |
| 26-30 | 6.3 | 10.3 | 14.1 | 11.4 | 9.1 |
| >30 | 13.0 | 13.0 | 14.7 | 13.7 | 12.1 |
| **Level of health facility** | | | | | |
| I (health centre) | 6.0 | 0.7 | 1.2 | 1.7 | 1.6 |
| II (hospital) | 49.7 | 51.4 | 41.1 | 46.8 | 46.7 |
| III (specialised hospital) | 44.3 | 47.9 | 57.7 | 51.6 | 51.8 |
| **Time from training to death certification+** | | | | | |
| < 3 months | - | - | 26.0 | 26.0 | - |
| 3-6 months | - | - | 50.6 | 50.6 | - |
| >6 months | - | - | 23.4 | 23.4 | - |

^+^ Does not include certificates from the pre-intervention study group.
